# Tang-Luo-Ning Improves Mitochondrial Antioxidase Activity in Dorsal Root Ganglia of Diabetic Rats: A Proteomics Study

**DOI:** 10.1155/2017/8176089

**Published:** 2017-01-04

**Authors:** Taojing Zhang, Yanbin Gao, Yanbin Gong, Hui Zhou, Peifeng Xie, Song Guan, Wenming Yi

**Affiliations:** ^1^Department of Endocrinology, Dongfang Hospital, Beijing University of Chinese Medicine, Beijing 100078, China; ^2^School of Traditional Chinese Medicine, Capital Medical University, Beijing 100069, China

## Abstract

Tang-luo-ning (TLN) is a traditional Chinese herbal recipe for treating diabetic peripheral neuropathy (DPN). In this study, we investigated mitochondrial protein profiles in a diabetic rat model and explored the potential protective effect of TLN. Diabetic rats were established by injection of streptozocin (STZ) and divided into model, alpha lipoic acid (ALA), and TLN groups. Mitochondrial proteins were isolated from dorsal root ganglia and proteomic analysis was used to quantify the differentially expressed proteins. Tang-luo-ning mitigated STZ-induced diabetic symptoms and blood glucose level, including response time to cold or hot stimulation and nerve conductive velocity. As compared to the normal, there were 388 differentially expressed proteins in the TLN group, 445 in ALA group, and 451 in model group. As compared to the model group, there were 275 differential proteins in TLN group and 251 in ALA group. As compared to model group, mitochondrial complex III was significantly decreased, while glutathione peroxidase and peroxidase were increased in TLN group. When compared with ALA group, the mitochondrial complex III was increased, and mitochondrial complex IV was decreased in TLN group. Together, TLN should have a strong antioxidative activity, which appears to be modulated through regulation of respiratory complexes and antioxidases.

## 1. Introduction

Diabetic peripheral neuropathy (DPN) is a microvascular complication of diabetes mellitus associated with pain, numbness, and dysfunction of motor and autonomic nervous system, which seriously affects the quality of life of these patients [1]. DPN is thought to play a role in the pathogenesis of diabetic foot. Diabetic patients with DPN have a 3- to 5-fold higher risk of developing diabetic foot when compared with patients without DPN [2, 3]. Incomplete understanding of the pathogenesis of DPN has largely been responsible for the lack of development of effective therapeutic modalities.

Several studies have implicated oxidative stress, especially that resulting from mitochondrial insult, as being the prime event in the pathogenesis of DPN [4, 5]. The role of oxidative stress resulting from mitochondrial injury has garnered much attention in the context of several diseases, including DPN. When blood glucose levels are high, oxidative stress is generated within mitochondria during energy production.

Mitochondrial membrane potential is widely used as a standard to determine the mitochondrial function [6]. A host of enzymes are involved in the mitochondrial metabolism, including respiratory complexes and antioxidases [7]. A balance between these two types of enzyme systems sustains mitochondrial function. However, the specific mitochondrial proteins that are affected in diabetes mellitus are not known. This study focused on the analysis of mitochondrial proteins using proteomics to investigate the pathogenesis of DPN and to study the mechanism underlying the protective effect of traditional Chinese herbal recipe, Tang-luo-ning (TLN).

Tang-luo-ning is comprised of* Astragalus* root,* Fructus corni*,* Rhizomacibotii*, and* Salvia miltiorrhiza*. We previously reported that the general efficacy of treatment with TLN was 91.67% in DNP patients [8], when used prior to oral mecobalamin treatment. Moreover, TLN treatment appeared to ameliorate DNP-related signs and symptoms, including numbness, pain, muscle spasm, and impairment of ankle reflex. Additional observed effects of TLN included improved nerve conduction velocity and peripheral microcirculation and enhanced sorbitol content in the red blood cells [8–10]. In animal studies, TLN appears to protect against the progression of DPN through inhibition of oxidative damage and downregulation of apoptosis in the dorsal root ganglion and sciatic nerve [11]. However, whether mitochondrial proteins are affected in diabetic model or as a result of TLN treatment is not known.

In this study, an experimental animal model for diabetes mellitus was developed and proteomics method was employed to study the alterations in mitochondrial protein profiles. Further, we explored the potential protective effect of TLN in restoring the expression of mitochondrial proteins.

## 2. Materials and Methods

### 2.1. Diabetic Model and Treatment

Eight-week-old male Sprague Dawley (SD) rats (Clean grade, 200–250 g, *n* = 75) were obtained from Vital River Laboratories (China, SCXK [Jing] 2012-0001). The rats were bred at the Animal Center of the Beijing University of Chinese Medicine (China) as per the National Standards (Gb14925_2001) [11]. The ambient environment was maintained at a temperature of 25 ± 1°C and 60 ± 10% humidity. Fourteen rats formed the control group and the remaining 61 rats were used to produce diabetic model. Diabetes mellitus model was induced by intraperitoneal injection of 60 mg/kg streptozocin (STZ). Blood glucose was measured after 72 h of injection; a value >16.7 mmol/L was considered indicative of the successful establishment of the diabetes model. A total of 61 diabetic rats were randomly divided into alpha lipoic acid (ALA) group (19 rats), TLN group (21 rats), and model group (21 rats). Rats without modeling were used as control group. Model group consisted of 21 rats in which diabetes was established by exposure to STZ but that received no further treatment. The study protocol was approved by the Ethics Committee at the Beijing University of Chinese Medicine. All animal experiments were conducted in accordance with the NIH guidelines for the care and use of laboratory animals (NIH Publication number 80-23; revised 1978) [11]. TLN were administrated to the rats of TLN group for 8 consecutive weeks. TLN was prepared at the Dongfang Hospital (No. 6, First District, Fangxingyuan, Fengtai District, Beijing City, China) at a concentration of 10 g/mL (crude drug). The ingredients of TLN included* Astragalus* root,* Fructus corni*,* Rhizomacibotii*, and* Salvia miltiorrhiza*. TLN was orally administrated at a dose of 5 g/kg/d (5 mL, 2.5 mg/mL) for 56 days. Similarly, in the alpha lipoic acid (ALA) group, ALA was administrated orally (2.5 mg/kg/d diluted in distilled water, 5 mL). A similar volume of distilled water was used in the model group. In addition, the effect of oral administration of ALA (diluted with distilled water) on mitochondrial protein profile was also measured.

### 2.2. Determination of Glycosylated Hemoglobin and Glucose Level

After treatment for 56 days, glycosylated hemoglobin was calculated following the instruction of the assay kit (Nanjing Jiancheng, Nanjing, China, Catalog number: A056).

Glucose level was determined every four weeks. For this assessment, caudal vein blood was collected after eight-hour diet.

### 2.3. Determination of Reaction Time to Heat and Cold Stimulation

The rats were fixed in a holder. The tail was immersed into water (43 ± 0.5°C or 10 ± 0.5°C) to test the reaction time to hot or cold stimulation.

### 2.4. Determination of Motor Nerve Conduction Velocity of Sciatic Nerve

The rats were exposed to 10% chloral hydrate (350 mg/kg, i.p.) for anesthesia and subsequently fixed in a prone position. A stimulating electrode was placed on the sciatic notch at the sciatic nerve efferent site. The reference electrode was placed between the stimulating electrode and the recording electrode. A single square wave pulse (width 0.1 ms) was delivered at stimulation intensity of 1.5-fold of the threshold with 5 s interval between two stimulations. The room temperature was set strictly at 20 ± 5°C. The time taken for the action potential to be transmitted from stimulation point to the distal muscle was recorded. Stimulation was repeated 7–10 times and mean values were calculated. Motor sciatic nerve conduction velocity (MNCV) was calculated: MNCV (m/s) = distance from stimulating electrode to recording electrode/latency.

### 2.5. Protein Digestion and Peptide Labeling

Eight weeks after drug treatment, L6, S1 dorsal root ganglion were obtained as described previously [12]. Mitochondria were isolated from the dorsal root ganglia. Mitochondrial proteins were isolated in different groups at a later stage. Briefly, DTT was added into the protein solution to a final concentration of 10 mM. After a 56°C water-bathing for 1 h, IAM was rapidly added into the solution to a final concentration of 55 mM in dark room for 1 h. Precooled acetone (4-fold volume to sample) was added to the solution. After deposition for at least 3 h in −20°C, the solution was centrifuged at 4°C at 20000 g for 20 min. The supernatant was discarded and deposition was diluted in 300 *μ*L rediluted buffer (50% TEAB, 0.1% SDS). After further centrifugation (4°C, 20000 g, 30 min), the supernatant was collected. After isolation, the protein concentration was measured by Bradford assay.

Proteins were digested by trypsin (1 *μ*g/*μ*L) at a concentration of 100 *μ*g substrate with 3.3 *μ*g enzyme at 37°C for 24 h. The digested material was cold-dried; peptides were dissolved in triethylammonium bicarbonate (TEAB) (H_2_O : TEAB: 1 : 1, 0.1% SDS) and 60 *μ*L peptide was included in each tube. The peptides were labeled by different isotopes using the iTRAQ method. Eight isotopes of similar volume were applied to label the digested peptides. After marking (control group, isotope 113-114; TLN group, isotope 115-116; ALA group, isotope 117-118; model group, isotope 119, 121), the labeled samples were dried and identified and quantified by MS/MS.

### 2.6. High-Performance Liquid Chromatography (HPLC)

**Figure 1:**
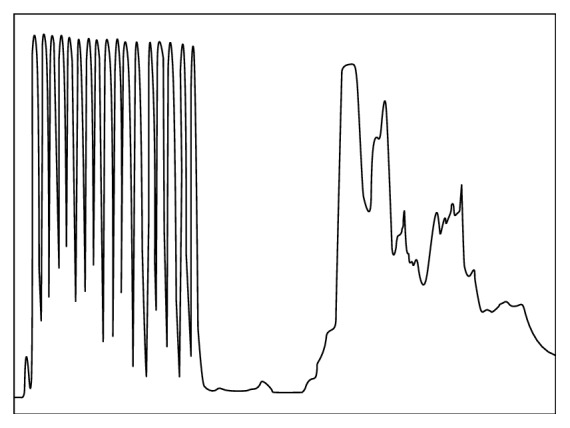
Isolation of SCX.

**Figure 2:**
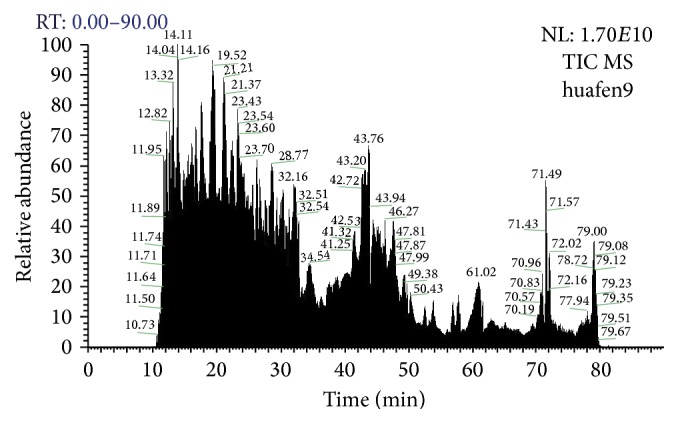
Q-Exactive mass spectrum.

Strong cation exchange (SCX) chromatography assisted the peptide isolation. Labeled samples were diluted in Solution A (25% CAN, 10 mM KH_2_PO_4_, pH 3.0). Under this condition, the peptides were positively charged and absorbed due to exchange with the metal ions. The later elution was completed by Solution B (25% ACN, 2 M KCl, 10 mM KH_2_PO_4_, pH 3.0). Solution A and solution B were filtered by 0.22 *μ*m and 0.45 *μ*m organic film. HPLC with strong cation exchange chromatography was applied to detect the samples. The isolation of SCX is shown in Figure 1. Peptide purification was completed by C18 reversed phase column chromatography. Peptide signals were detected by Q-Exactive mass spectrometer (MS), which is shown in Figure 2. MS signals were transferred to PD software (Proteome Discoverer 1.3, Thermo). Qualification and quantification were completed by Mascot software.

### 2.7. Statistical Analyses

All data are presented as mean ± standard deviation (SD). All statistical analyses were performed using SPSS 11.5. Least significant difference (LSD)* t*-tests were performed for intergroup differences. The differences among three groups or more were completed by one-way ANOVA followed by Tukey's test. A* P* value of <0.05 was considered as statistically significant.

## 3. Results

### 3.1. TLN Alleviated STZ-Induced Diabetic Symptoms

As shown in Table 1, STZ significantly elevated the level of glycosylated hemoglobin (*F* (3, 71) = 78.73, *P* < 0.0001). At eight and twelve weeks, STZ-induced increase of glucose level was mitigated by TLN, but not by ALA treatment (12 weeks:* F* (3, 71) = 68.33, *P* < 0.0001) (Table 2). Both TLN and ALA reduced STZ-induced increase of response time to hot and cold stimulation at eight- and twelve-week time points (12 weeks:* F* (3, 71) = 43.13, *P* < 0.0001) (Tables 3 and 4).

Nerve conduction velocity was also measured. As shown in Table 5, diabetic rats showed decrease in the conduction velocity, which was ameliorated by TLN treatment at each time point, while ALA only ameliorated the decrease at 4-week, 8-week, and 12-week time points (12 weeks:* F* (3, 71) = 25.33, *P* < 0.0001) (Table 5).

### 3.2. Quantitative Analysis of Mitochondrial Proteins

**Figure 3:**
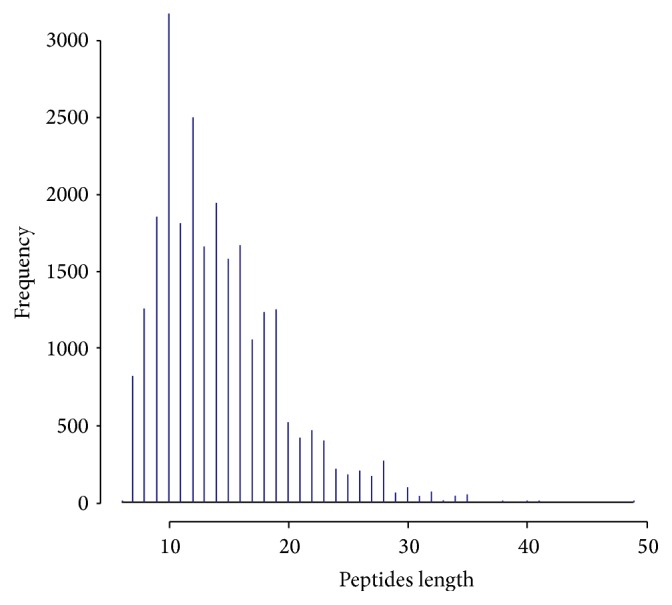
Distribution of peptide lengths in Uniprotrat library.

Using a standard curve, mitochondrial proteins were calculated in all study groups. The concentrations of mitochondrial proteins in model, normal, TLN, and western medicine groups were 1.1 *μ*g/*μ*L, 0.9 *μ*g/*μ*L, 1.2 *μ*g/*μ*L, and 1.4 *μ*g/*μ*L, respectively. The quantities of proteins in all the four groups were sufficient for carrying out the proteomics study. The lengths of the peptides are shown in Figure 3. Proteins in Uniprotrat library were quantified (Table 6). Differential proteins were defined by a direct ratio higher than 1.2 with an associated *P* value of <0.05.

As compared with the control group, there were 388 differential proteins (176 downregulated, 212 upregulated) in the TLN group, 445 (198 downregulated, 247 upregulated) in the western medicine group, and 451 (205 downregulated, 246 upregulated) in the model group. As compared with the model group, there were 275 differential proteins (129 downregulated, 146 upregulated) in the TLN group and 251 (149 downregulated, 102 upregulated) in the western medicine group. As compared to the TCM group, there were 250 differential proteins (106 downregulated, 144 upregulated) in the western medicine group.

### 3.3. Qualitative Analysis of Mitochondrial Proteins

The differentially expressed proteins are shown in Tables 7 [Table tab8] [Table tab9]–10. The levels of mitochondrial complexes and aconitase were significantly increased in the model group (*P* < 0.05), while antioxidases were significantly decreased (*P* < 0.05), as compared to that in the control group. These differences might serve to attenuate the antioxidative ability of mitochondria in the diabetic model. There was a significant decrease in mitochondrial complex III, with a concomitant significant increase in glutathione peroxidase and peroxidase in the TLN group as compared to that in the model group. As listed in Table 7, 11 mitochondrial proteins were influenced after diabetic modeling, whereas only four proteins (mitochondrial complex III cytochrome C reductase 2, mitochondrial complex III cytochrome C reductase 1, glutathione peroxidase, and peroxidase) were reversed after TLN treatment in diabetic rats (Table 9). Further, the mitochondrial complex III, glutathione peroxidase, and peroxidase were increased in the TLN group as compared to that in the western medicine group. The increase in expression of glutathione peroxidase and peroxidase was particularly marked in the TLN group.

## 4. Discussion

Although TLN continues to be employed for treatment of DPN patients in China, the underlying protective mechanisms are not well understood. In this study, we demonstrated that TLN may have a protective role against STZ-induced diabetic symptoms. Using proteomics method, we found that a series of mitochondrial proteins were affected in the diabetic rat model. Mitochondrial oxidases and mitochondrial complexes were altered in the dorsal root ganglia of DPN rats, possibly contributing to the decreased antioxidant effect. Further, TLN appeared to restore the expression of these proteins. The changes in mitochondrial proteins might contribute to injury associated with DPN in diabetic rat model, while the antioxidant effect of TLN could be attributed to the restoration of the mitochondrial proteins.

Proteomics is a large-scale study of the structure and function of proteins and their role in the progression and resolution of morbid changes in diseases. The proteomics method provides pervasive information for the target screening and discovery of potential cascades [13]. Using this method, alterations of protein expression can be screened. These differences might explain the pathogenesis of disease and help identify potential therapeutic targets. Proteomics has seen wide application in several recent studies [14]. Isobaric tag for relative and absolute quantification (iTRAQ) has been applied in proteomics in combination with tandem mass spectrometry [15, 16].

In the present study, we employed this method and used eight types of iTRAQ reagents (isotope reagents). After enzyme digestion, peptides were labeled by eight isotopes and were later accurately identified and quantified. A total of 903 mitochondrial proteins were screened. Under hyperglycemic conditions, the expression of approximately half of the mitochondrial proteins was found to be altered, which indicates that the mitochondrion is likely to be a key organelle affected in DPN.

A series of signaling pathways are known to be involved in death of dorsal root ganglion in DPN model [17]. Additionally, organelle stress such as endoplasmic reticulum stress has also been reported in diabetic animal models [18]. As the energy generation organelle, mitochondria play a key role in the generation of free radical oxidative stress (ROS). Impairment of mitochondrial respiratory chain or mitochondrial membrane could potentially lead to generation of ROS, thereby inducing the activation of apoptotic signaling cascades [19]. However, the mitochondrial protein expression profile in diabetic model or that after drug treatment was not known. In the present study, we found that, in a STZ-induced diabetes model, half of the mitochondrial proteins were either downregulated or upregulated as compared to that in the control group. There are mainly two types of mitochondrial proteins: mitochondrial complexes and antioxidases [20, 21]. Mitochondrial complexes are responsible for energy generation, while antioxidases are responsible for the clearance of oxidative free radicals. A state of homeostasis between these two types of enzymes is essential for sustenance of mitochondrial function. In the present study, the levels of mitochondrial complexes and aconitase in the model group were significantly increased, while antioxidases were significantly decreased. This imbalance could have been the cause of impaired mitochondrial function. On further analysis, 451 proteins appeared to be affected in the diabetic model, including upregulation of 246 proteins and downregulation of 205 proteins, which is indicative of the severity of mitochondrial insult. Among the 451 proteins, several oxidative stress-related proteins such as aconitase, glutathione peroxidase, uncoupling protein 2, peroxidase, and manganese superoxide dismutase, were affected. In contrast, mitochondria complexes were upregulated. The bidirectional dysregulation appears to have been responsible for the impaired mitochondrial function. Precisely, 11 mitochondrial proteins were severely affected after diabetic modeling, including mitochondrial complex III cytochrome C reductase 2, aconitase, mitochondrial complex I NADH-coenzyme Q reductase 5, mitochondrial complex IV cytochrome C oxidase subunit 2, mitochondrial complex III cytochrome C reductase 1, mitochondrial complex II succinate dehydrogenase complex subunit A, mitochondrial complex IV cytochrome C oxidase subunit 1, glutathione peroxidase, uncoupling protein 2, peroxidase, and manganese superoxide dismutase. Among them, only four proteins (mitochondrial complex III cytochrome C reductase 2, mitochondrial complex III cytochrome C reductase 1, glutathione peroxidase, and peroxidase) were reversed after TLN treatment in diabetic rats. These four proteins might be the therapeutic targets for TLN. However, further experiments are required to confirm our hypothesis.

Alpha lipoic acid is the most common first-line antioxidant therapy for DPN [22, 23]. In this study, we found that the expression of 251 mitochondrial proteins was affected after ALA administration, including 149 proteins that were upregulated and 102 proteins that were downregulated. These findings indicate that the antioxidative effect of ALA in the diabetic model was through regulation of mitochondrial proteins. Interestingly, TLN altered the expression of 275 proteins when compared with the model group. These findings suggest a stronger antioxidative effect of TLN than ALA.

The antioxidant capacity of the ingredients of TLN has been demonstrated in earlier studies. For example,* Astragalus* root was shown to reduce the serum glucose and triglyceride levels and inhibit advanced glycation end products-induced inflammation in diabetic individuals [24, 25]. Astragaloside IV has also been shown to protect against DPN in STZ-induced diabetes in rats [26].* Fructus corni* or its extract is reported to protect against diabetes-induced oxidative stress, inflammation, and advanced glycation end products in the livers of STZ-induced diabetic rats [27, 28]. Finally,* Rhizomacibotii* is reported to enhance ATP genesis [29]. Based on these studies, the strong antioxidative ability of TLN appears to be due to the different antioxidant components. Further, these different components might have an additive effect in ameliorating the oxidative stress. It is also possible that different components target different enzymes, which leads to a strong antioxidant effect. Although antioxidative proteins were found after modeling and TLN treatment in this study, further confirmation is necessary.

## 5. Conclusion

In this study, we employed proteomics to study the altered expression of multiple mitochondrial proteins in the dorsal root ganglia in a diabetic rat model. TLN appeared to have a strong antioxidant effect possibly by regulating the expression of mitochondrial respiratory complexes and antioxidases. These results provide a novel explanation for the antioxidant effect of TLN. Although several mitochondrial proteins were found to be differentially regulated in the different treatment groups, their functional effects could not be investigated in detail on account of limitation of time and funding. We hope to study these aspects in greater detail in the future.

## Figures and Tables

**Table 1:** TLN decreased glycosylated hemoglobin level in diabetic model. Data are presented as mean and SD.

Groups	Glycosylated hemoglobin
Control	6.76 ± 1.55
Model	14.98 ± 2.18^△△^
TLN	12.69 ± 1.09^△△*∗*^
ALA	13.85 ± 1.44^△△^

^△△^ *P* < 0.01 compared to control group; ^*∗*^ *P* < 0.05 compared with model group.

**Table 2:** TLN decreased blood glucose level in diabetic model. Data are presented as mean and SD.

Groups	Glucose level (mmol/L)			
	0 weeks	4 weeks	8 weeks	12 weeks
Control	5.43 ± 0.72	5.49 ± 0.66	5.52 ± 0.59	5.71 ± 0.55
Model	26.85 ± 3.22^△△^	25.23 ± 2.96^△△^	26.11 ± 2.35^△△^	24.67 ± 3.97^△△^
TLN	26.05 ± 3.32	24.41 ± 3.17	22.01 ± 2.79^*∗*#^	19.31 ± 2.69^*∗∗*#^
ALA	24.71 ± 4.54	24.33 ± 3.12	23.99 ± 2.37	23.87 ± 2.80

^△△^ *P* < 0.01 compared to control group; ^*∗*^ *P* < 0.05, ^*∗∗*^ *P* < 0.01 compared with model group; ^#^ *P* < 0.05 compared with ALA group.

**Table 3:** TLN ameliorated STZ-induced decrease of response time to hot stimulation. Data are presented as mean and SD.

Groups	Response time to hot stimulation (s)			
	0 weeks	4 weeks	8 weeks	12 weeks
Control	24 ± 12	20 ± 17	27 ± 11	26 ± 15
Model	11 ± 8^△△^	37 ± 12^△^	88 ± 32^△△^	77 ± 29^△△^
TLN	15 ± 18	41 ± 21	51 ± 17^*∗∗*^	49 ± 34^*∗∗*^
ALA	13 ± 13	42 ± 19	55 ± 31^*∗∗*^	51 ± 23^*∗∗*^

^△^ *P* < 0.05, ^△△^ *P* < 0.01 compared to control group; ^*∗∗*^ *P* < 0.01 compared with model group.

**Table 4:** TLN ameliorated STZ-induced decrease of response time to cold stimulation. Data are presented as mean and SD.

Groups	Response time to cold stimulation (s)			
	0 weeks	4 weeks	8 weeks	12 weeks
Control	20 ± 11	23 ± 15	25 ± 14	24 ± 13
Model	21 ± 10	27 ± 16	79 ± 21^△△^	84 ± 26^△△^
TLN	16 ± 16	28 ± 19	64 ± 29^*∗*^	61 ± 28^*∗∗*#^
ALA	17 ± 12	30 ± 24	62 ± 27^*∗*^	78 ± 22

^△△^ *P* < 0.01 compared to control group; ^*∗*^ *P* < 0.05, ^*∗∗*^ *P* < 0.01 compared with model group; ^#^ *P* < 0.05 compared with ALA group.

**Table 5:** TLN ameliorated STZ-induced decrease of nerve conductive velocity. Data are presented as mean and SD.

Groups	Nerve conductive velocity (m/s)			
	0 weeks	4 weeks	8 weeks	12 weeks
Control	53.34 ± 4.39	57.66 ± 3.66	54.68 ± 3.17	55.71 ± 3.99
Model	44.81 ± 3.78^△△^	43.77 ± 3.14^△△^	40.18 ± 5.12^△△^	39.25 ± 2.75^△△^
TLN	47.88 ± 3.74^*∗*^	48.75 ± 4.15^*∗∗*^	51.33 ± 2.88^*∗∗*^	53.14 ± 4.78^*∗∗*#^
ALA	45.53 ± 2.88	46.22 ± 3.75^*∗*^	48.41 ± 3.27^*∗∗*^	49.39 ± 2.88^*∗∗*^

^△△^ *P* < 0.01 compared to control group; ^*∗*^ *P* < 0.05, ^*∗∗*^ *P* < 0.01 compared with model group; ^#^ *P* < 0.05 compared with ALA group.

**Table 6:** The list of Uniprotrat library.

Total spectrum	563136
Matched spectrum	25048
Peptides	3677
Matched proteins	903

**Table 7:** Protein ratio of control group to model group.

Protein number	Names	Ratio	*P *value
Q6AY12	Mitochondrial complex III cytochrome C reductase 2	0.439	0.0013
P23764	Aconitase	0.473	0.0019
Q06QA1	Mitochondrial complex I NADH- coenzyme Q reductase 5	0.572	0.0021
G3CH61	Mitochondrial complex IV cytochrome C oxidase subunit 2	0.606	0.0039
G3V9S0	Mitochondrial complex III cytochrome C reductase 1	0.673	0.0037
Q0QF18	Mitochondrial complex II succinate dehydrogenase complex subunit A	0.76	0.0153
B0FT61	Mitochondrial complex IV cytochrome C oxidase subunit 1	0.775	0.0231
Q6PDW8	Glutathione peroxidase	1.781	0.0029
D3ZIA7	Uncoupling protein 2	1.912	0.0031
P62963	Peroxidase	1.987	0.0027
Q9QXQ0	Manganese superoxide dismutase	2.542	0.0014

**Table 8 tab8:** Protein ratio of ALA group to model group.

Protein number	Names	Ratio	*P*
G3V9S0	Mitochondrial complex III cytochrome C reductase 1	0.758	0.0244
Q6AY12	Mitochondrial complex III cytochrome C reductase 2	0.826	0.0329

**Table 9 tab9:** Protein ratio of TLN group to model group.

Protein number	Names	Ratio	*P*
Q6AY12	Mitochondrial complex III cytochrome C reductase 2	0.806	0.0314
G3V9S0	Mitochondrial complex III cytochrome C reductase 1	0.811	0.0407
Q6PDW8	Glutathione peroxidase	1.305	0.0311
P62963	Peroxidase	1.549	0.0284

**Table 10:** Protein ratio of TLN group to ALA group.

Protein number	Names	Ratio	*P*
G3CH61	Mitochondrial complex IV cytochrome C oxidase subunit 2	0.73	0.0271
G3V9S0	Mitochondrial complex III cytochrome C reductase 1	1.244	0.0384
Q6PDW8	Glutathione peroxidase	1.273	0.0294
P62963	Peroxidase	1.354	0.0277
